# Association of a multimodal perioperative nutritional/ERAS care pathway with postoperative infectious complications following radical hysterectomy for cervical cancer: a retrospective nonconcurrent cohort study

**DOI:** 10.3389/fpubh.2026.1950549

**Published:** 2026-09-30

**Authors:** Keke Zhang, Qian Chen, Rongrong Cai

**Affiliations:** 1 Department of Gynecology, Jinhua Central Hospital, Jinhua, Zhejiang, China; 2 Department of Operating Room, Huai'an Hospital of Huai'an City (Huai'an Clinical Medical College of Jiangsu University), Huai'an, Jiangsu, China; 3 Department of Gynecology, Nantong Haimen District People's Hospital, Nantong, Jiangsu, China

**Keywords:** cervical cancer, infectious complications, length of hospital stay, perioperative nutritional support, radical hysterectomy

## Abstract

**Background:**

Malnutrition is prevalent in women with cervical cancer and may be associated with postoperative infectious complications following radical hysterectomy. Evidence specifically evaluating structured multimodal perioperative nutritional/ERAS care in this population remains limited.

**Methods:**

We conducted a single-center retrospective nonconcurrent cohort study of 268 patients who underwent radical hysterectomy for cervical cancer (FIGO stages IA–IIB) between January 2020 and December 2024. Patients who received a structured multimodal perioperative nutritional/ERAS care pathway (NSG, *n* = 134) were compared with patients receiving standard care (CG, *n* = 134). Nutritional status was assessed using NRS-2002 and serial albumin, prealbumin, and CRP measurements. The primary outcome was any postoperative infectious complication within 30 days. Multivariable logistic regression was used to examine associations with infection risk.

**Results:**

Baseline characteristics were similar in magnitude for measured variables; however, nonsignificant baseline tests were not interpreted as evidence of equivalence or successful matching. The 30-day infectious complication rate was lower in the NSG than in the CG (18.7% vs. 33.6%; unadjusted RR 0.56, 95% CI 0.36–0.85; *p* = 0.005). SSI was also less frequent (6.0% vs. 14.2%; *p* = 0.026), whereas the difference in UTI did not reach conventional statistical significance (8.2% vs. 15.7%; *p* = 0.060). By POD 7, albumin was 39.1 ± 4.0 versus 34.2 ± 4.9 g/L, and mean LOS was 12.3 ± 4.1 versus 15.8 ± 5.6 days. In the exploratory multivariable model, pathway exposure was associated with lower adjusted odds of infectious complications (adjusted OR 0.47; 95% CI 0.26–0.85; *p* = 0.013).

**Conclusion:**

Receipt of the structured multimodal perioperative nutritional/ERAS care pathway was associated with lower observed odds of 30-day infectious complications and shorter hospitalization. Because the study used a retrospective nonconcurrent comparison and the intervention was bundled, these findings should not be interpreted as proof that nutritional support alone caused the observed differences. Prospective comparative studies are warranted.

## 1 Introduction

Cervical cancer ranks as the fourth most common cancer and the fourth leading cause of cancer-related death in women worldwide, with an estimated 660,000 new cases and 350,000 deaths reported in 2022 (1, 2). The burden is disproportionately concentrated in low- and middle-income countries (LMICs), where access to preventive interventions and early detection programs remains limited. Despite the advent of prophylactic HPV vaccination and organized screening programs, cervical cancer continues to impose substantial morbidity and mortality, particularly among women aged 35–55 years in high-incidence regions of sub-Saharan Africa, South Asia, and Latin America (1, 2). In China, cervical cancer is the most common gynecological malignancy, with an estimated 120,000 new cases annually, and the dual burden of high disease prevalence and increasing surgical case volumes makes optimization of perioperative outcomes a pressing public health priority (3).

Radical hysterectomy with pelvic lymph node dissection is a technically demanding procedure associated with a wide spectrum of intraoperative and postoperative complications beyond the well-recognized infectious risks (4). Non-infectious complications include urological injuries, lower limb lymphedema, bladder dysfunction, and vascular injury, each of which contributes to prolonged recovery and reduced health-related quality of life (4). Infectious complications, however, represent the most frequent and potentially modifiable class of morbidity: surgical site infections (SSI) impair fascial healing and may necessitate prolonged wound care or reoperation, urinary tract infections (UTI) are amplified by mandatory postoperative catheterization, and pulmonary complications are compounded by the catabolic effects of major pelvic surgery on respiratory muscle function (5, 6). Collectively, postoperative infectious events prolong hospitalization by an estimated 3–7 days, substantially increase direct medical costs, and may delay initiation of adjuvant therapy—a clinically consequential effect in patients with high-risk pathological features (4).

Among the modifiable predictors of postoperative infectious complications after radical hysterectomy, nutritional status has attracted growing clinical and research attention. It is estimated that 20–50% of cancer patients experience clinically significant malnutrition at the time of surgery, with cervical cancer patients facing a unique nutritional challenge due to tumor-related anorexia, metabolic reprogramming secondary to chronic inflammation, and the pronounced catabolic stress of neoadjuvant chemotherapy when administered prior to surgery (7, 8). Preoperative malnutrition—as reflected by low serum albumin, impaired cellular immune function, reduced muscle mass, and attenuated antioxidant capacity—creates a biological substrate for increased susceptibility to perioperative infection, impaired wound healing, prolonged ileus, and extended hospital stay (9, 10). The postoperative period further amplifies nutritional vulnerability through elevated catabolic hormone secretion, restricted oral intake, and the inflammatory response to surgical trauma, creating a self-reinforcing cycle of protein depletion and immune suppression (7).

Nutritional and inflammation-related indices have demonstrated prognostic associations in cervical and other gynecologic cancers. Meta-analyses have shown that a low Prognostic Nutritional Index (PNI), calculated from serum albumin and peripheral lymphocyte count, is associated with poorer survival and adverse clinicopathological features in cervical and other gynecologic cancers (11, 12). In early-stage cervical cancer with high-risk factors, the preoperative Controlling Nutritional Status (CONUT) score has also been reported as a prognostic factor (13). These indices support the broader clinical relevance of nutritional assessment, although they should not be interpreted as direct evidence that perioperative nutritional intervention prevents postoperative infection. The Nutritional Risk Screening 2002 (NRS-2002) tool is a validated screening instrument developed for identifying hospitalized patients at nutritional risk (14).

Evidence from adjacent gynecologic oncology and other cancer-surgery settings suggests that perioperative nutrition and immunonutrition may influence postoperative outcomes, although the evidence is heterogeneous. A study in ovarian cancer evaluated the clinical and immunological impact of immunonutrition (15), while postoperative immune-modulating diets were associated with fewer wound complications in gynecologic oncology patients undergoing laparotomy (16). A 2024 systematic review of perioperative immunonutrition in gynecological cancer included six studies involving 712 patients; four studies reported fewer postoperative infections or complications, but intervention timing, formulations, and study designs varied substantially (17). In upper gastrointestinal cancer surgery, a meta-analysis of randomized trials found that perioperative immunonutrition reduced infectious complications (RR 0.72, 95% CI 0.57–0.92) (18). These findings provide supportive context but are not directly equivalent to radical hysterectomy for cervical cancer.

The ERAS (Enhanced Recovery After Surgery) Society recommendations for gynecologic oncology describe a multimodal perioperative pathway that includes evidence-based measures intended to accelerate recovery (5). The 2023 ERAS Society update specifically focused on implementation challenges and barriers to adoption of ERAS recommendations rather than constituting a nutrition-specific guideline update (6). Nutritional screening and perioperative nutrition remain supported by dedicated ESPEN guidance for cancer and surgical patients (7, 8). Accordingly, the present study evaluates a bundled nutritional/ERAS-aligned pathway and does not attribute observed outcomes to any single nutritional component. Nutritional risk screening should be distinguished from the diagnosis of malnutrition, for which the GLIM consensus provides a framework (19).

From a mechanistic perspective, several components of perioperative nutritional and ERAS-aligned care may plausibly influence postoperative recovery, including preoperative nutritional repletion, carbohydrate loading, early enteral intake, and coordinated dietitian oversight. However, albumin, prealbumin, CRP, infection, and recovery outcomes are influenced by multiple clinical factors, and the effects of individual components cannot be isolated when they are delivered as a bundled pathway. Therefore, mechanistic explanations in the present study are treated as hypotheses rather than demonstrated causal pathways.

This study aimed to examine whether receipt of a structured multimodal perioperative nutritional/ERAS care pathway was associated with the incidence of 30-day postoperative infectious complications and length of hospital stay among patients undergoing radical hysterectomy for cervical cancer at a single tertiary center in China. Secondary objectives were to explore associations between available clinical and nutritional variables and postoperative infection, describe perioperative biomarker trajectories, and conduct exploratory subgroup analyses. Given the retrospective nonconcurrent design, all findings were interpreted as associations rather than causal effects.

## 2 Methods

### 2.1 Study design and setting

We conducted a single-center retrospective nonconcurrent cohort study at Jinhua Central Hospital covering January 2020 to December 2024. The study received institutional ethics approval (Approval No. 2023KY023) and was conducted in accordance with the Declaration of Helsinki. The approval covered the retrospective review of the complete study cohort. Because perioperative practice evolved during the study period and the structured pathway was implemented within routine clinical care, calendar-time and secular-trend confounding are important design limitations.

### 2.2 Study population

The study population comprised women diagnosed with cervical cancer who underwent elective radical hysterectomy (Wertheim-Meigs procedure) with bilateral pelvic lymph node dissection during the study period. The final analytic cohort contained 134 patients in the structured-care group and 134 in the control group. No formal matching procedure was performed. All eligible patients meeting the inclusion criteria were included according to pathway exposure, and baseline characteristics are presented descriptively (Figure 1).

**Figure 1:**
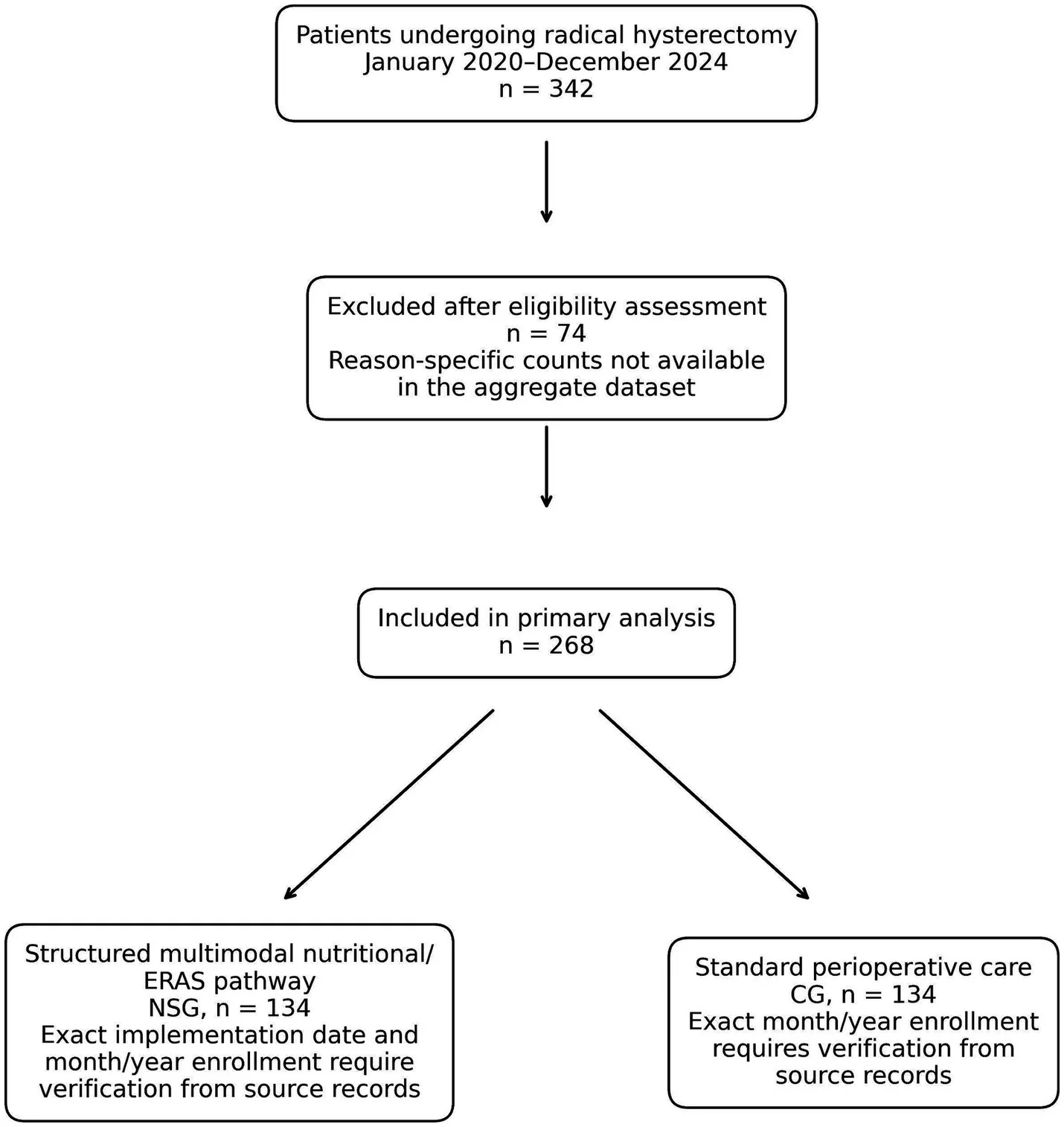
Study flow diagram depicting patient screening, eligibility assessment, group allocation, and inclusion in the primary analysis.

Inclusion criteria: (1) histologically confirmed cervical cancer; (2) FIGO 2018 staging IA–IIB; (3) elective radical hysterectomy as primary or definitive surgery; (4) age ≥18 years; and (5) complete perioperative nutritional and laboratory records.

Exclusion criteria: (1) previous pelvic or abdominal surgery within 6 months; (2) FIGO stage III–IV; (3) concurrent immunosuppressive therapy; (4) concurrent autoimmune or inflammatory bowel disease; (5) incomplete clinical records; and (6) concurrent non-cervical malignancy.

### 2.3 Multimodal perioperative nutritional/ERAS care pathway

The structured pathway comprised several coordinated perioperative components and should therefore be interpreted as a bundled nutritional/ERAS-aligned intervention rather than nutritional supplementation alone. The contribution of individual components cannot be separated in this observational study. The protocol included: (1) preoperative NRS-2002 screening at admission; patients with NRS-2002 ≥ 3 received oral nutritional supplementation (400–600 kcal/day and ≥18 g protein/day) for 7–14 days before surgery plus dietary counseling; all NSG patients received a 200 mL maltodextrin carbohydrate drink 2 h before surgery; (2) intraoperative goal-directed fluid management; and (3) postoperative early oral fluids within 4–6 h, with enteral tube feeding within 24 h when patients were unable to meet ≥60% of caloric requirements orally, plus dietitian assessment on POD 1, 3, and 5. Control patients received the standard perioperative care used during the corresponding treatment period. Detailed component-level comparisons were not available because the intervention was implemented as a bundled pathway. Because the intervention was bundled, the effects of individual components cannot be separated in this observational comparison.

### 2.4 Outcome measures

The primary outcome was the occurrence of any postoperative infectious complication within 30 days of surgery, including SSI, UTI, pneumonia, abdominal/pelvic abscess, bacteremia/sepsis, and lymphocyst infection. Outcomes were retrospectively identified from clinical records. SSI was classified using CDC/NHSN criteria. Other infectious diagnoses were abstracted from the clinical record as documented during routine care; this retrospective ascertainment is acknowledged as a potential source of misclassification. Complications were graded using the Clavien-Dindo classification (20). Patients could experience more than one infection type, so category-specific infection counts may exceed the number of patients with any infection.

Secondary clinical outcomes included length of hospital stay (LOS), time to first flatus, 30-day readmission, and total hospital cost. Time to first oral feeding and time to ambulation were considered process/recovery measures because they may be directly influenced by the broader perioperative pathway. Nutritional biomarkers (albumin, prealbumin, and CRP) were measured preoperatively and on POD 1, 3, 5, and 7. Body weight at discharge was interpreted cautiously because the measurement time depended on LOS.

### 2.5 Statistical analysis

Between-group comparisons for continuous variables presented as mean ± SD used two-sided independent-samples *t* tests. Categorical variables were compared using Pearson chi-square tests; Fisher’s exact test (or its exact extension for R × C tables) was used when expected cell counts were small. Effect estimates are presented with 95% confidence intervals where appropriate. No *a priori* sample-size calculation was performed for this retrospective analysis; statistical uncertainty is therefore emphasized through confidence intervals. The exploratory multivariable logistic regression model reported in Table 1 included eight fitted predictors: structured pathway exposure, NRS-2002 ≥ 3, diabetes mellitus, operation time ≥240 min, intraoperative blood loss ≥500 mL, neoadjuvant chemotherapy, preoperative anemia, and open surgical approach. With 70 primary-outcome events, this corresponds to approximately 8.8 events per fitted predictor; adjusted estimates are therefore interpreted cautiously. Repeated biomarker measurements are presented descriptively by time point; pointwise *p* values are unadjusted, exploratory, and do not account for within-patient correlation or multiplicity. Secondary-outcome and subgroup *p* values are also unadjusted for multiplicity and are interpreted as exploratory. Subgroup interaction tests used logistic models containing group, subgroup, and group-by-subgroup terms. All tests were two-tailed and *p* < 0.05 was considered statistically significant for the primary outcome. Analyses used SPSS version 27.0 and R version 4.3.1.

**Table 1:** Exploratory logistic regression analysis of factors associated with postoperative infectious complications.

Variable	Univariate analysis			Multivariate analysis		
	Crude OR	95% CI	*p*	Adj. OR	95% CI	*p*
Structured perioperative pathway (yes vs. no)	0.45	0.26–0.80	0.006	0.47	0.26–0.85	0.013
NRS-2002 ≥ 3 (vs. < 3)	4.74	2.58–8.70	<0.001	2.31	1.26–4.22	0.007
Diabetes mellitus (yes vs. no)	2.37	1.16–4.83	0.018	1.98	0.92–4.26	0.079
Operation time ≥240 min (vs. <240 min)	2.34	1.31–4.18	0.004	2.18	1.20–3.96	0.011
Intraoperative blood loss ≥500 mL (vs. <500 mL)	2.12	1.09–4.13	0.027	1.87	0.95–3.68	0.071
Neoadjuvant chemotherapy (yes vs. no)	2.85	1.55–5.24	0.001	1.65	0.84–3.24	0.146
Preoperative anemia (yes vs. no)	1.87	1.04–3.36	0.036	1.72	0.94–3.14	0.078
Open surgery (vs. laparoscopic)	1.36	0.79–2.36	0.272	1.42	0.79–2.55	0.239
Age ≥60 years (vs. <60 years)	1.34	0.72–2.51	0.354	—	—	—
BMI ≥ 25 kg/m^2^ (vs. <25 kg/m^2^)	1.23	0.68–2.22	0.492	—	—	—

OR: odds ratio; CI: confidence interval; Adj. OR: adjusted odds ratio; NRS-2002: Nutritional Risk Screening 2002. Crude ORs are univariate logistic-regression estimates. The multivariable model included the eight predictors shown with adjusted estimates in the table. There were 70 primary-outcome events, corresponding to approximately 8.8 events per fitted predictor; therefore, the adjusted model is exploratory and may be sensitive to model specification. Residual confounding, particularly by calendar time and other unmeasured clinical factors, cannot be excluded.

## 3 Results

### 3.1 Patient flow and baseline characteristics

A total of 342 patients underwent radical hysterectomy during the study period. After application of the eligibility criteria, 268 patients were included: 134 in the NSG and 134 in the CG. Baseline values of the measured characteristics were similar in magnitude (Table 2); however, nonsignificant *p* values were not interpreted as evidence that the groups were equivalent or formally matched.

**Table 2:** Baseline and perioperative characteristics of study participants.

Variable	NSG (*n* = 134)	CG (*n* = 134)	*p*-value
Demographics			
Age (years), mean ± SD	48.3 ± 9.7	49.1 ± 10.3	0.513
BMI (kg/m^2^), mean ± SD	22.7 ± 3.4	23.2 ± 3.7	0.250
Tumor characteristics			
FIGO stage, *n* (%)			0.963
IA	8 (6.0%)	7 (5.2%)	
IB	78 (58.2%)	75 (56.0%)	
IIA	34 (25.4%)	37 (27.6%)	
IIB	14 (10.4%)	15 (11.2%)	
Histological subtype, *n* (%)			0.966
Squamous cell carcinoma	105 (78.4%)	103 (76.9%)	
Adenocarcinoma	25 (18.7%)	27 (20.1%)	
Adenosquamous carcinoma	4 (3.0%)	4 (3.0%)	
Nutritional status			
NRS-2002 score, *n* (%)			0.714
<3 (low risk)	72 (53.7%)	69 (51.5%)	
≥3 (at risk)	62 (46.3%)	65 (48.5%)	
Preoperative albumin (g/L), mean ± SD	38.2 ± 4.8	37.9 ± 5.1	0.620
Preoperative prealbumin (mg/L), mean ± SD	228.4 ± 48.3	225.7 ± 51.2	0.657
Preoperative hemoglobin (g/L), mean ± SD	112.3 ± 18.7	110.8 ± 19.4	0.520
Comorbidities			
Diabetes mellitus, *n* (%)	18 (13.4%)	20 (14.9%)	0.726
Hypertension, *n* (%)	28 (20.9%)	31 (23.1%)	0.658
Preoperative anemia, *n* (%)	42 (31.3%)	45 (33.6%)	0.696
Treatment factors			
Neoadjuvant chemotherapy, *n* (%)	29 (21.6%)	31 (23.1%)	0.769
Surgical approach, *n* (%)			0.807
Open (laparotomy)	68 (50.7%)	70 (52.2%)	
Laparoscopic/robotic	66 (49.3%)	64 (47.8%)	
Operation time (min), mean ± SD	215.4 ± 48.7	219.8 ± 52.3	0.477
Intraoperative blood loss (mL), mean ± SD	287.3 ± 156.8	294.6 ± 168.2	0.714

NSG: structured-care group; CG: control group; BMI: body mass index; NRS-2002: Nutritional Risk Screening 2002; SD: standard deviation. Continuous variables were compared using two-sided independent-samples *t* tests. Categorical variables were compared using Pearson chi-square tests; the histological-subtype comparison used the Fisher–Freeman–Halton exact test because of small expected cell counts. Baseline *p* values are descriptive only and were not used to claim equivalence or successful matching.

### 3.2 Perioperative nutritional biomarkers

Baseline nutritional biomarker levels were similar between groups. Descriptively, albumin and prealbumin values were higher and CRP values lower in the NSG from POD 3 onward. By POD 7, mean albumin was 39.1 ± 4.0 g/L in the NSG and 34.2 ± 4.9 g/L in the CG. Body weight loss at discharge was smaller in the NSG (−2.3 ± 2.1% vs. −4.8 ± 3.2%). Because these were repeated measurements and the available table reports pointwise comparisons, the p values should be interpreted as exploratory rather than as a definitive longitudinal treatment effect (Table 3; Figure 2).

**Table 3:** Perioperative nutritional status indicators by group and time point.

Indicator	Time point	NSG (*n* = 134)	CG (*n* = 134)	*p*-value
Albumin (g/L)	Preoperative	38.2 ± 4.8	37.9 ± 5.1	0.620
	POD 1	31.4 ± 4.2	30.8 ± 4.6	0.266
	POD 3	33.8 ± 4.1	31.2 ± 4.8	<0.001
	POD 5	36.7 ± 4.3	32.4 ± 5.0	<0.001
	POD 7	39.1 ± 4.0	34.2 ± 4.9	<0.001
Prealbumin (mg/L)	Preoperative	228.4 ± 48.3	225.7 ± 51.2	0.657
	POD 1	162.3 ± 38.4	158.7 ± 41.2	0.460
	POD 3	188.5 ± 42.1	162.4 ± 45.8	<0.001
	POD 5	215.8 ± 44.7	172.3 ± 48.9	<0.001
	POD 7	241.2 ± 46.8	185.6 ± 52.3	<0.001
CRP (mg/L)	Preoperative	8.3 ± 6.7	8.7 ± 7.1	0.636
	POD 1	68.4 ± 28.7	72.1 ± 31.4	0.315
	POD 3	42.8 ± 22.3	58.7 ± 29.8	<0.001
	POD 5	24.3 ± 14.8	45.6 ± 25.7	<0.001
	POD 7	12.7 ± 8.4	32.4 ± 18.9	<0.001
Body weight change at discharge (%)		−2.3 ± 2.1	−4.8 ± 3.2	<0.001

POD: postoperative day; CRP: C-reactive protein; NSG: structured-care group; CG: control group. Values are mean ± SD. Pointwise p values were calculated using two-sided independent-samples *t* tests and are exploratory because they do not account for within-patient correlation or multiplicity; a longitudinal mixed-effects model would be preferable for confirmatory inference.

**Figure 2:**
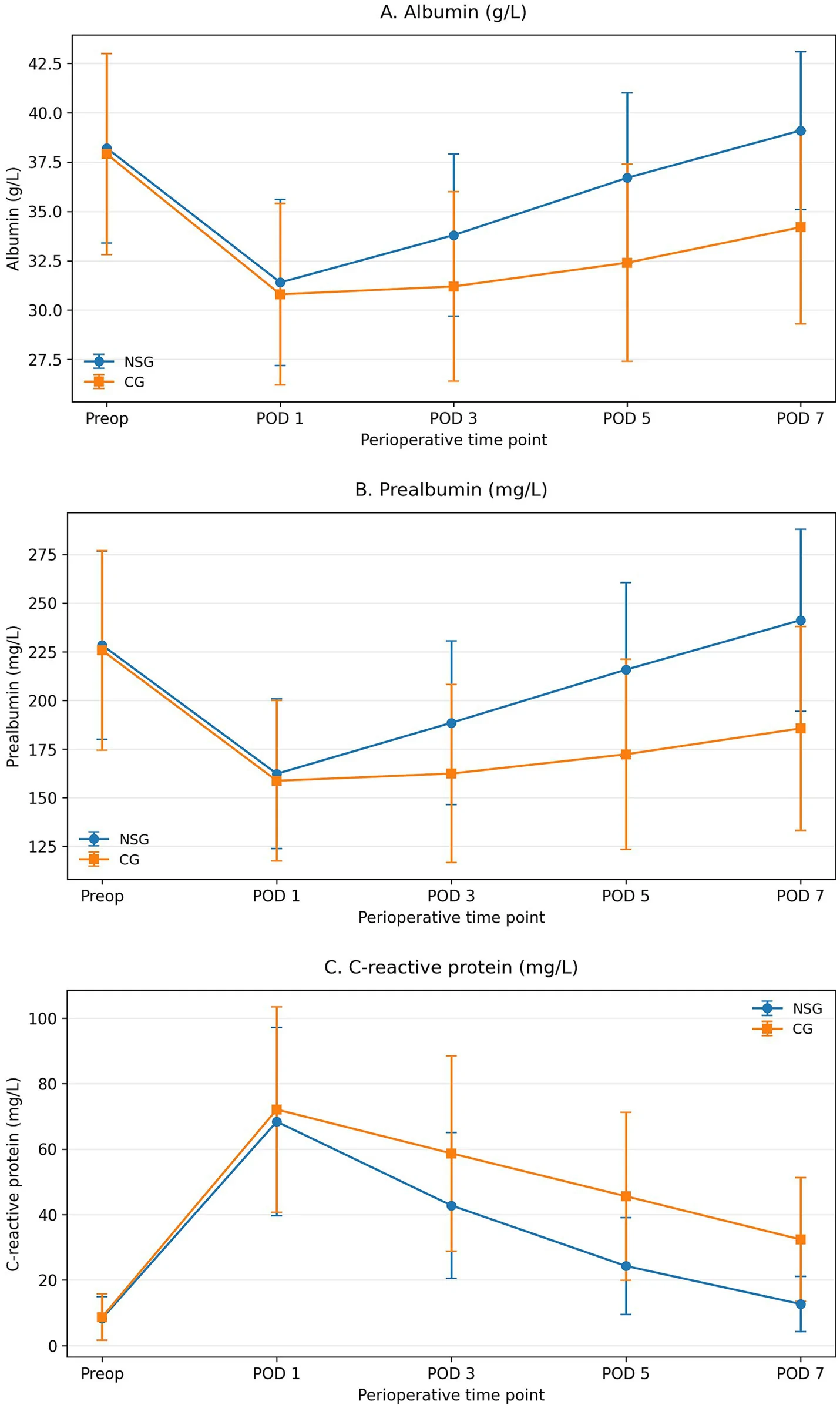
Descriptive changes in serum albumin **(A)**, prealbumin **(B)**, and C-reactive protein **(C)** across perioperative time points. Error bars should be interpreted together with the corresponding summary statistics. Pointwise between-group p values are exploratory because repeated measurements within patients are correlated and no multiplicity-adjusted longitudinal model is presented.

### 3.3 Postoperative infectious complications

The primary outcome of any 30-day infectious complication occurred in 25/134 patients (18.7%) in the NSG and 45/134 (33.6%) in the CG (Pearson chi-square *p* = 0.005). This corresponds to an absolute risk difference of −14.9 percentage points (95% CI − 25.3 to −4.6) and an unadjusted risk ratio of 0.56 (95% CI 0.36–0.85). SSI occurred in 6.0% versus 14.2% (unadjusted RR 0.42, 95% CI 0.19–0.93; *p* = 0.026). UTI was numerically less frequent in the NSG (8.2% vs. 15.7%), but the difference was not statistically significant (unadjusted RR 0.52, 95% CI 0.26–1.04; *p* = 0.060). Prolonged postoperative antibiotic use was treated as a process/treatment measure rather than as an infectious complication (Table 4; Figure 3).

**Table 4:** Postoperative infectious complications within 30 days.

Outcome/measure	NSG (*n* = 134)	CG (*n* = 134)	*p*-value
Any infectious complication	25 (18.7%)	45 (33.6%)	0.005
Surgical site infection (SSI), any	8 (6.0%)	19 (14.2%)	0.026
Superficial SSI	5 (3.7%)	11 (8.2%)	0.122
Deep incisional SSI	3 (2.2%)	8 (6.0%)	0.124
Urinary tract infection	11 (8.2%)	21 (15.7%)	0.060
Pneumonia	5 (3.7%)	10 (7.5%)	0.184
Abdominal/pelvic abscess	3 (2.2%)	7 (5.2%)	0.197
Bacteremia/sepsis	2 (1.5%)	5 (3.7%)	0.447
Lymphocyst infection	4 (3.0%)	8 (6.0%)	0.237
Antibiotic use >5 postoperative days (process measure)	18 (13.4%)	38 (28.4%)	0.003
Clavien-Dindo ≥ II complications	16 (11.9%)	33 (24.6%)	0.007

NSG: structured-care group; CG: control group. SSI classified by CDC/NHSN criteria. Patients could experience more than one infection type. Pearson chi-square tests were used for the displayed categorical comparisons except Fisher’s exact test for bacteremia/sepsis because of small expected counts. Prolonged antibiotic use is a treatment/process measure and is shown separately from infection diagnoses. Except for the prespecified primary outcome (‘any infectious complication’), category-specific p values are unadjusted for multiplicity and should be interpreted as exploratory.

**Figure 3:**
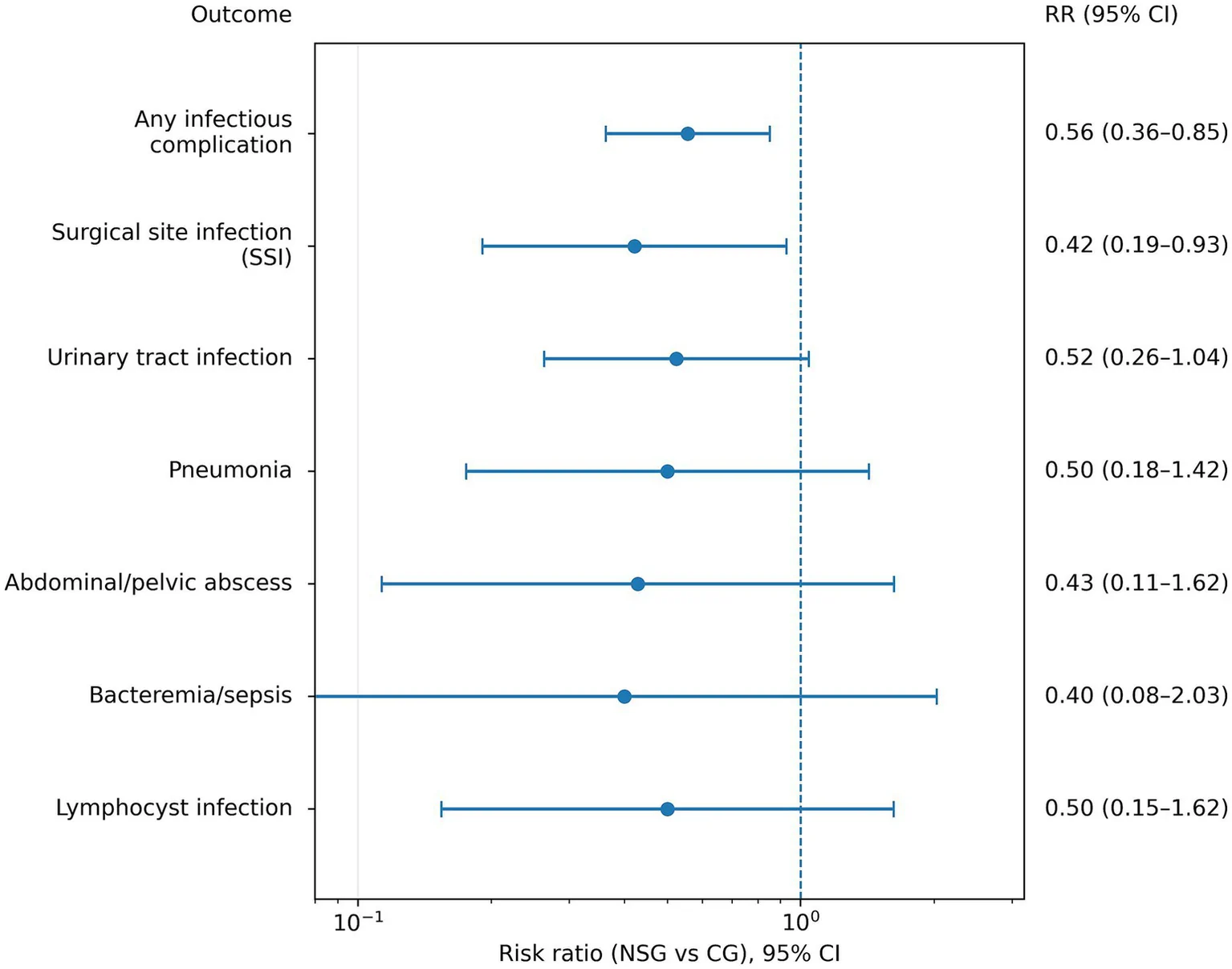
Unadjusted risk ratios (RRs) and 95% confidence intervals for 30-day postoperative infectious complications in the structured-care group (NSG) versus the control group (CG). Points represent effect estimates and horizontal lines represent 95% confidence intervals. The dashed vertical line indicates RR = 1.

### 3.4 Postoperative recovery and clinical outcomes

Several recovery and utilization measures differed between groups (Table 5). Mean LOS was 12.3 ± 4.1 days in the NSG versus 15.8 ± 5.6 days in the CG, corresponding to a mean difference of −3.5 days (95% CI − 4.68 to −2.32; *p* < 0.001). Time to first flatus, oral feeding, and ambulation were shorter in the NSG, although oral feeding and ambulation are partly protocol-dependent process measures and should not be interpreted as independent clinical effects. Total recorded hospital cost was lower in the NSG (52.4 ± 12.8 vs. 63.7 ± 18.4 × 10^3^ CNY); because costs were recorded across 2020–2024 and were not standardized to a common price year, the economic comparison remains exploratory.

**Table 5:** Postoperative clinical outcomes.

Outcome	NSG (*n* = 134)	CG (*n* = 134)	*p*-value
Primary recovery endpoints			
Length of hospital stay (days)	12.3 ± 4.1	15.8 ± 5.6	<0.001
Time to first flatus (hours)	28.4 ± 8.7	36.8 ± 11.3	<0.001
Time to first oral feeding (hours)	18.7 ± 6.4	32.5 ± 10.8	<0.001
Time to first ambulation (hours)	24.3 ± 7.8	31.2 ± 9.4	<0.001
Safety and serious events			
ICU admission, *n* (%)	5 (3.7%)	11 (8.2%)	0.122
Unplanned reoperation, *n* (%)	1 (0.7%)	3 (2.2%)	0.622
30-day hospital readmission, *n* (%)	4 (3.0%)	10 (7.5%)	0.100
Postoperative bladder dysfunction, *n* (%)	42 (31.3%)	48 (35.8%)	0.438
Lower limb lymphedema, *n* (%)	15 (11.2%)	18 (13.4%)	0.577
30-day mortality, *n* (%)	0 (0%)	1 (0.7%)	1.000
Resource utilization			
Total hospital cost (×10^3^ CNY), mean ± SD	52.4 ± 12.8	63.7 ± 18.4	<0.001

NSG: structured-care group; CG: control group; ICU: intensive care unit; CNY: Chinese Yuan. Continuous outcomes were compared using two-sided independent-samples *t* tests. Categorical outcomes were compared using Pearson chi-square tests, with Fisher’s exact test for unplanned reoperation and 30-day mortality because of small expected counts. Time to oral feeding and ambulation are pathway-sensitive process/recovery measures. Hospital costs are nominal amounts and were not standardized to a common price year; the cost comparison and all secondary-outcome p values are exploratory and unadjusted for multiplicity.

### 3.5 Exploratory multivariable association analysis

In the exploratory multivariable logistic regression model, receipt of the structured perioperative pathway was associated with lower adjusted odds of postoperative infectious complications (adjusted OR 0.47; 95% CI 0.26–0.85; *p* = 0.013). NRS-2002 ≥ 3 (adjusted OR 2.31; 95% CI 1.26–4.22; *p* = 0.007) and operation time ≥240 min (adjusted OR 2.18; 95% CI 1.20–3.96; *p* = 0.011) were associated with higher adjusted odds. These estimates should be interpreted cautiously because the model cannot eliminate residual confounding, including temporal changes in care (Table 1).

### 3.6 Subgroup analyses

Exploratory subgroup results are shown in Table 6. The stratum-specific odds ratios were generally below 1.0, but formal interaction tests did not provide evidence of statistically significant effect modification. In particular, the interaction p value for NRS-2002 status was 0.990 in the unadjusted interaction model, rather than the previously reported value of 0.047. Given the small subgroup sample sizes and observational design, these analyses are hypothesis-generating only.

**Table 6:** Exploratory subgroup analysis of the association between the structured perioperative pathway and infectious complications.

Subgroup	NSG, *n*/*N* (%)	CG, *n*/*N* (%)	OR (95% CI)	Interaction *p*
By NRS-2002 score				
NRS-2002 < 3	6/72 (8.3%)	12/69 (17.4%)	0.43 (0.15–1.22)	0.990
NRS-2002 ≥ 3	19/62 (30.6%)	33/65 (50.8%)	0.43 (0.21–0.89)	
By diabetes status				
With diabetes mellitus	5/18 (27.8%)	11/20 (55.0%)	0.31 (0.08–1.22)	0.561
Without diabetes mellitus	20/116 (17.2%)	34/114 (29.8%)	0.49 (0.26–0.92)	
By FIGO stage				
Stage IA–IB	12/86 (14.0%)	23/82 (28.0%)	0.42 (0.19–0.90)	0.736
Stage IIA–IIB	13/48 (27.1%)	22/52 (42.3%)	0.51 (0.22–1.17)	
By surgical approach				
Open (laparotomy)	14/68 (20.6%)	26/70 (37.1%)	0.44 (0.20–0.94)	0.895
Laparoscopic/robotic	11/66 (16.7%)	19/64 (29.7%)	0.47 (0.20–1.10)	
By neoadjuvant chemotherapy				
Received NACT	9/29 (31.0%)	17/31 (54.8%)	0.37 (0.13–1.07)	0.684
No NACT	16/105 (15.2%)	28/103 (27.2%)	0.48 (0.24–0.96)	

OR: odds ratio; CI: confidence interval; NSG: structured-care group; CG: control group; NACT: neoadjuvant chemotherapy; NRS-2002: Nutritional Risk Screening 2002. Stratum-specific ORs and 95% CIs are unadjusted Wald estimates derived from the displayed 2 × 2 counts. Interaction p values were estimated from unadjusted logistic models containing group, subgroup, and group-by-subgroup terms. All subgroup analyses are exploratory, and no statistically significant interaction was identified.

## 4 Discussion

In this retrospective nonconcurrent cohort of 268 patients undergoing radical hysterectomy for cervical cancer, receipt of a structured multimodal perioperative nutritional/ERAS care pathway was associated with lower observed odds of 30-day infectious complications and a 3.5-day shorter mean hospital stay. The adjusted OR of 0.47 corresponds to approximately 53% lower adjusted odds, not a 53% reduction in risk. Based on the observed proportions, the absolute risk difference was −14.9 percentage points (95% CI − 25.3 to −4.6), and the unadjusted risk ratio was 0.56 (95% CI 0.36–0.85). The LOS mean difference was −3.5 days (95% CI − 4.68 to −2.32). These findings are consistent with prior literature linking nutritional status and perioperative care with postoperative outcomes; however, the present observational design does not establish causality and residual confounding cannot be excluded. Sehouli et al. evaluated 226 women in a prospective gynecologic oncology cohort and reported associations between nutritional/body-composition measures and postoperative morbidity, providing relevant contextual evidence rather than a directly comparable intervention effect (9).

Approximately 47% of the cohort had NRS-2002 ≥ 3, and this variable was associated with higher adjusted odds of infection in the exploratory model. This finding is compatible with previous evidence that malnutrition and hypoalbuminemia are markers of higher perioperative risk (21). However, albumin is influenced by inflammation, fluid status, hepatic synthesis, vascular permeability, transfusion, and exogenous albumin administration and should not be interpreted as a specific measure of nutritional recovery. The postoperative divergence in albumin and prealbumin values therefore supports a difference in biomarker trajectories but does not by itself demonstrate attenuation of protein catabolism.

CRP values also differed descriptively between groups after POD 3. Because CRP is a nonspecific acute-phase marker and may itself be affected by postoperative infection, the direction of any mechanistic relationship cannot be established from these data. Carbohydrate loading, early enteral intake, fluid management, and other pathway components could each influence inflammatory and recovery profiles. Accordingly, potential mechanisms involving insulin resistance, gut-barrier integrity, and immune function should be regarded as biologically plausible hypotheses rather than demonstrated mediators in this study.

The intervention evaluated here was a bundled perioperative pathway rather than isolated nutritional supplementation. This distinction is important because NRS-2002 screening, oral supplementation, carbohydrate loading, goal-directed fluid management, early oral intake, conditional enteral feeding, and dietitian assessment may each affect outcomes. Prior gynecologic oncology literature, including systematic evidence on perioperative immunonutrition, supports continued investigation of nutritional strategies, but differences in intervention composition and study design limit direct comparison (17, 22). The current findings therefore add institution-level observational evidence for a multimodal pathway while not isolating the contribution of nutrition alone. Standardized tools are recommended for nutritional screening and assessment (23, 24). An association between malnutrition and postoperative morbidity has also been reported in a national gynecologic cancer cohort (25).

The subgroup analyses should be interpreted cautiously. Although stratum-specific odds ratios generally favored the structured-care group, none of the formal interaction tests was statistically significant after correction of the previously inconsistent NRS-2002 interaction result. The small numbers within several strata further limit precision. These results are therefore exploratory and should not be used to claim that any particular subgroup derives a greater causal benefit.

Several limitations require emphasis. First, the retrospective nonconcurrent design precludes causal inference and is vulnerable to residual confounding. Calendar-time (secular-trend) confounding is a major concern because pathway exposure was associated with treatment period and perioperative practice may have evolved during 2020–2024, including changes in surgical experience, minimally invasive techniques, catheter management, antimicrobial prophylaxis, infection surveillance, nursing practice, discharge criteria, and broader ERAS implementation. Calendar year was not included in the current multivariable model; therefore, part of the observed association between the structured-care pathway and postoperative outcomes may reflect secular changes in care rather than the pathway itself. Second, the intervention was bundled, so the contribution of nutritional supplementation cannot be separated from other perioperative-care components. Third, the exploratory multivariable model contained eight fitted predictors for 70 primary-outcome events (approximately 8.8 events per predictor), did not include all clinically important potential confounders, and may therefore be vulnerable to overfitting, model-specification sensitivity, and residual confounding. Fourth, repeated biomarker values were compared at individual time points rather than with a full longitudinal mixed-effects model; these pointwise tests, as well as secondary-outcome and subgroup tests, were not adjusted for multiplicity and are interpreted as exploratory. Fifth, outcome ascertainment was retrospective, and detailed standardized diagnostic criteria were not prospectively specified for every infection category. Sixth, body weight was measured at discharge, which occurred at different postoperative times because LOS differed. Seventh, nominal hospital costs across multiple calendar years were not standardized to a common price year. Finally, the single-center setting and predominantly Han Chinese population may limit generalizability.

Future studies should use prospective multicenter designs with prespecified exposure definitions, contemporaneous comparator groups, standardized infection ascertainment, detailed protocol-adherence data, and *a priori* confounder selection. Calendar time and surgical team should be incorporated into the analytic strategy when relevant, and propensity-score or other design-based approaches may help reduce measured confounding. Longitudinal mixed-effects models are preferable for serial biomarkers, and cost analyses should account for price year and skewed distributions. More detailed measurement of actual energy/protein delivery, fluid balance, transfusion, albumin administration, renal/hepatic function, catheter and drain duration, and other perioperative factors would also improve mechanistic interpretation and reproducibility.

## 5 Conclusion

In this retrospective nonconcurrent cohort, receipt of a structured multimodal perioperative nutritional/ERAS care pathway was associated with lower observed odds of 30-day infectious complications and shorter hospitalization after radical hysterectomy for cervical cancer. Nutritional risk at admission was also associated with postoperative infectious morbidity. Because temporal confounding, residual confounding, bundled intervention effects, and retrospective outcome ascertainment cannot be excluded, the findings should be considered hypothesis-generating and should be confirmed in prospective comparative studies before attributing the observed differences to nutritional support alone or recommending causal practice changes.

## Data Availability

The datasets generated and/or analyzed during the current study are available from the corresponding author upon reasonable request.
